# Kite String Injury Causing a Complete Tear of the Tendoachilles

**DOI:** 10.7759/cureus.11827

**Published:** 2020-12-01

**Authors:** Nishant Bhatia, Akash Goel, Dharam Pal Swami, Shashank Rousa, Jaikaran Singh

**Affiliations:** 1 Department of Orthopaedic Surgery, Maulana Azad Medical College and Associated Lok Nayak Hospital, New Delhi, IND

**Keywords:** complete tear, kite string, krackow, manja, tendoachilles

## Abstract

Kite flying is a common activity in many countries, particularly India. Fierce competition has led to the use of dangerous kite threads like manja (which is a cotton or nylon thread coated with powdered glass) to gain an edge over rivals. The sharp manja can not only cause linear abrasions or deep incised wounds among flyers but it can inflict equally serious injuries to onlookers or unwary pedestrians and two-wheeler riders on the street. Hand, throat, angle of mouth, nose, and feet are some common sites of injury. We report a case of a 62-year-old man who sustained a manja cut injury through the tendoachilles while walking on the roadside when his foot got entangled in the barely visible thin thread as a bicycle rushed past him. The wound was debrided and tendoachilles repaired using the Krackow technique. The patient had reasonable strength in plantar-flexion and good active range of motion at the ankle when last seen at the one-year follow-up. The aim of this article is to highlight the hazards of manja and the need to revisit and acknowledge the clinical, social, and administrative implications of this unusual but noteworthy mechanism of limb and life-threatening injury.

## Introduction

Kite flying is a common activity in many countries, particularly in India, many parts of South America, and South-East Asia. Makar Sankranti (also known as Uttarayan) is the festival of kites, which is celebrated in Western parts of India (especially in the states of Gujrat, Maharashtra, and Rajasthan) in early January. Initially, kite flying was just a hobby but over the years, children and adults have started many competitions where the sole objective is to bring down the kite of their competitors by the clashing of strings. This has led to the use of more dangerous kite strings like ‘Manja,’ which gives them an advantage over their opponent. Manja is a type of kite thread made from cotton or nylon that is coated with finely powdered glass using chemical adhesives [1]. Flyers, unwary passers-by, and two-wheeler drivers especially in the populated areas can get injured due to this fine but very sharp thread [1]. The hand is the most common site of injury to the flyer, whereas the neck, angle of the mouth, bridge of the nose, and the forehead, near the hairline are common sites in pedestrians and two-wheeler riders [1]. Linear abrasions and incised wounds are the usual patterns of injury thought to occur due to a fast-moving thread cutting through the skin either while flying a kite or when a moving person accidentally entangles a limb, neck, or torso while coming across it [1].

The Achilles tendon is commonly ruptured by indirect force resulting from sudden loading of a plantar-flexed ankle especially while running on an incline or jumping [2]. Direct trauma resulting in tendoachilles rupture itself is uncommon while a kite string causing injury to this tendon and that too a complete transection is an extremely rare phenomenon [3].

After a thorough literature search, we came across only two cases whereby the reported cause of tendoachilles rupture was a kite string injury [3], however, the laceration was not complete in both of them, and in fact, the injury was missed in one of the cases, which later presented as a chronic rupture. The uniqueness of the case that we report here lies in the fact that the patient had a complete rupture of the tendon with clean-cut edges as if someone had incised it transversely with a very sharp knife. Luckily, the posterior tibial artery had escaped the injury. This pattern of injury, where a kite string was able to completely cut the tendoachilles while sparing the posterior tibial artery, has not been reported in the literature to the best of our knowledge.

The main aim of this article is to highlight this unique and rare but attention-worthy mechanism of injury. Even though the resulting pathology and its treatment options are fairly common in routine day-to-day practice, the etiology deserves a special mention because the injury it can inflict is easily missed by an unsuspecting eye and, moreover, there are several medico-legal, social, and administrative implications associated with it.

## Case presentation

A 62-year-old male presented to our orthopedic emergency department with a lacerated wound on the posterior aspect of the right ankle in January 2019. The mechanism of injury was narrated as a kite string (manja) getting entangled around his ankle while he was walking on the side of the road, unaware of any thread, and a bicycle rode speedily past him. The patient was unable to bear weight on the affected limb and was brought on a trolley.

**Figure 1:**
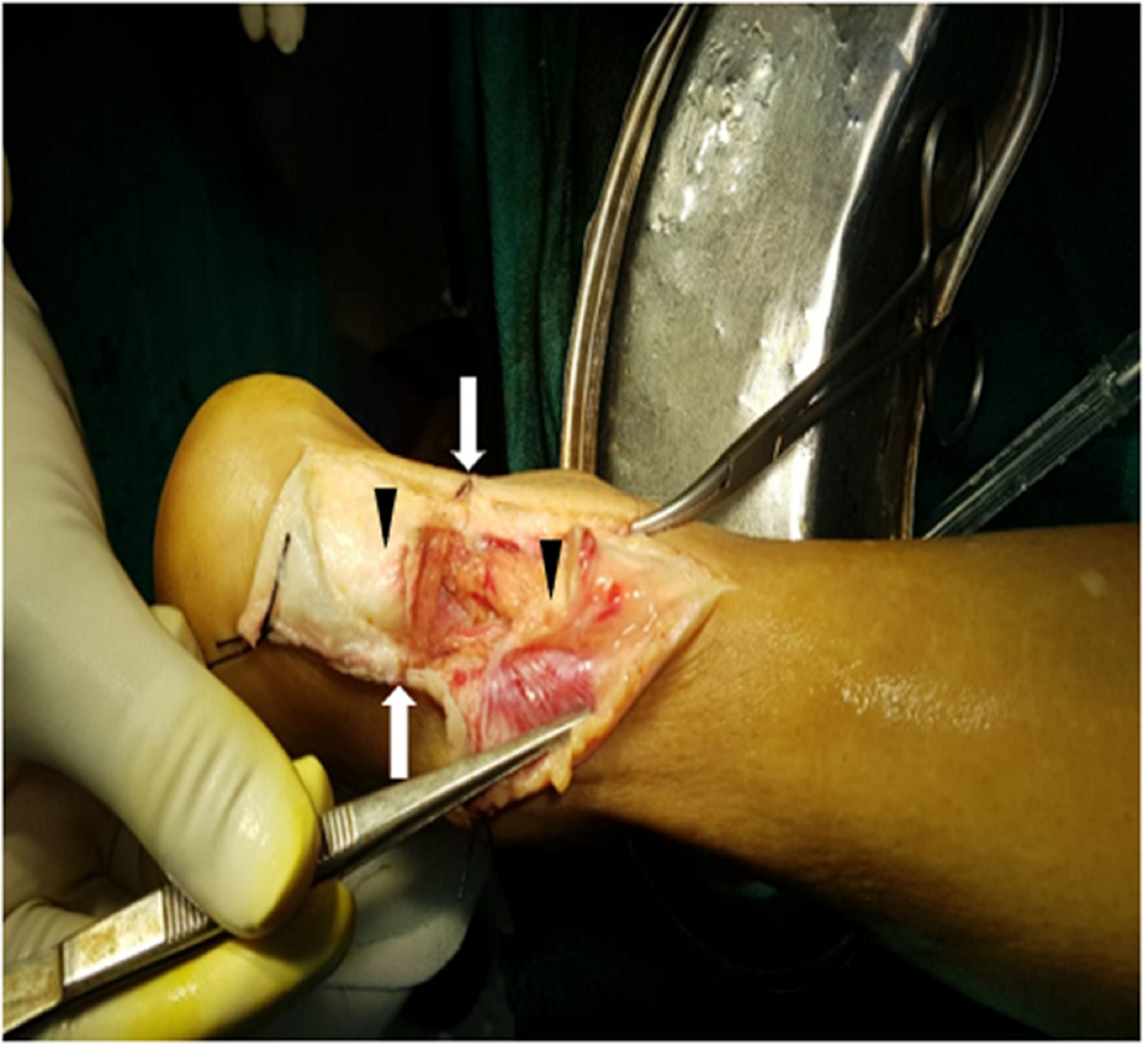
The severed tendoachilles The white arrows mark the extent of the original transverse incised wound. The wound was incorporated into the incision of the posteromedial approach followed by the raising of subfascial flaps. The black arrowheads point to the proximal and distal cut ends of the tendon.

Local examination revealed a clean-cut transverse lacerated wound of 5-cm x 2-cm size over the posterior aspect of the right ankle and a complete tear of the tendoachilles with both its cut ends visible through the wound (Figure 1). The corners of the wound had linear marks of the thread and were stained with its color. The Thompson test, Matles test, and O’Brien Needle test were positive. Active plantar-flexion at the ankle was not possible. Sensory and vascular examination of the foot was normal. Standard radiographs of the ankle did not reveal any bony abnormality.

**Figure 2:**
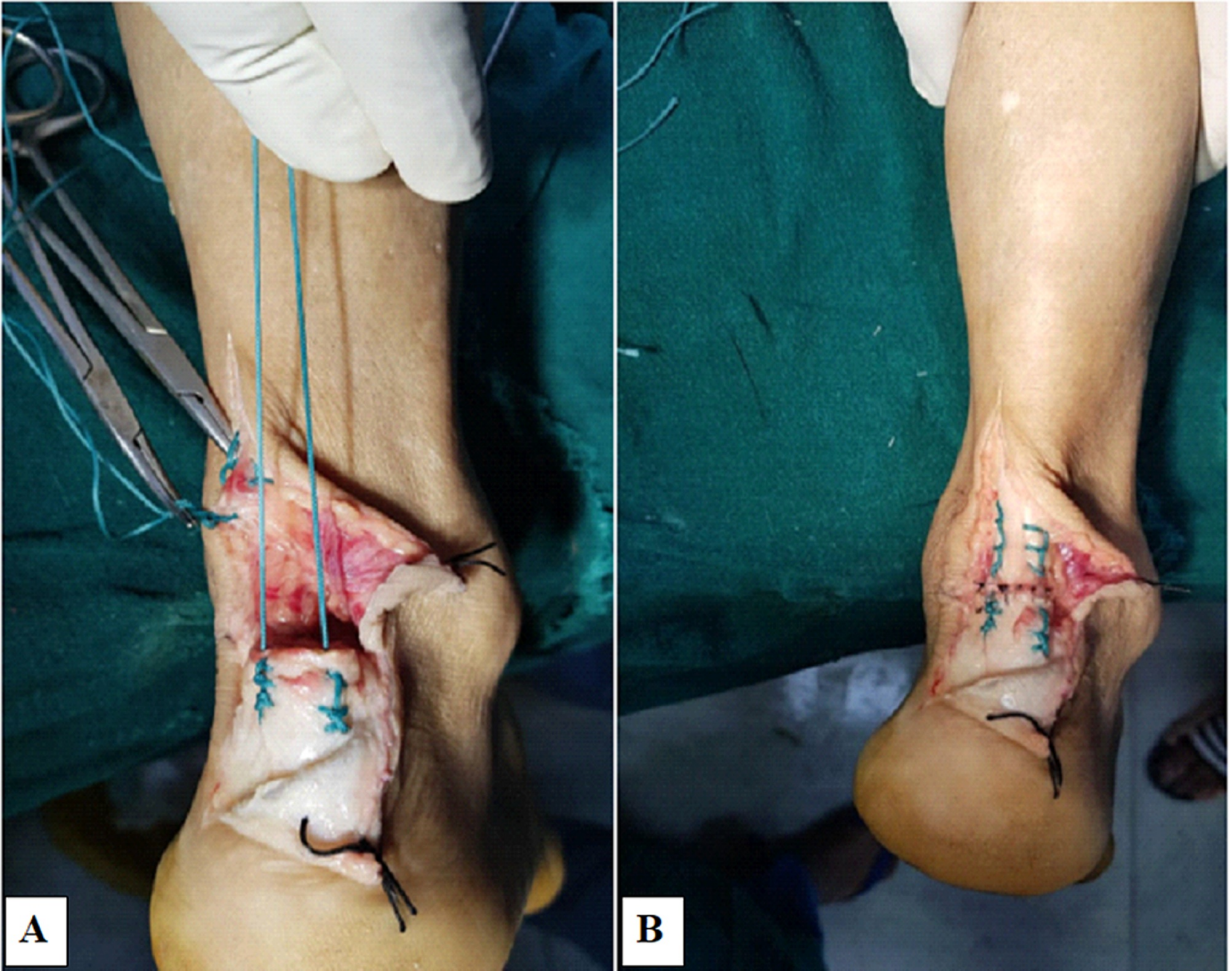
Primary repair of the tendon (A) Number five Ethibond suture used to perform the repair via the Krackow technique; (B) Completed repair with paratenon sutured

Tetanus prophylaxis was given, and antibiotics were started. Primary repair of the tendoachilles was performed under spinal anesthesia and tourniquet control with the patient in a prone position. An 8-cm-long, longitudinal, posteromedial skin incision was utilized that incorporated the wound margins also. All other vital structures were found to be intact after wound extension. Lavage, debridement, and gentle freshening of the margins was done. The repair was completed using a number five Ethibond suture (Ethicon Inc., Somerville, New Jersey) via the Krackow technique (Figure 2).

**Figure 3:**
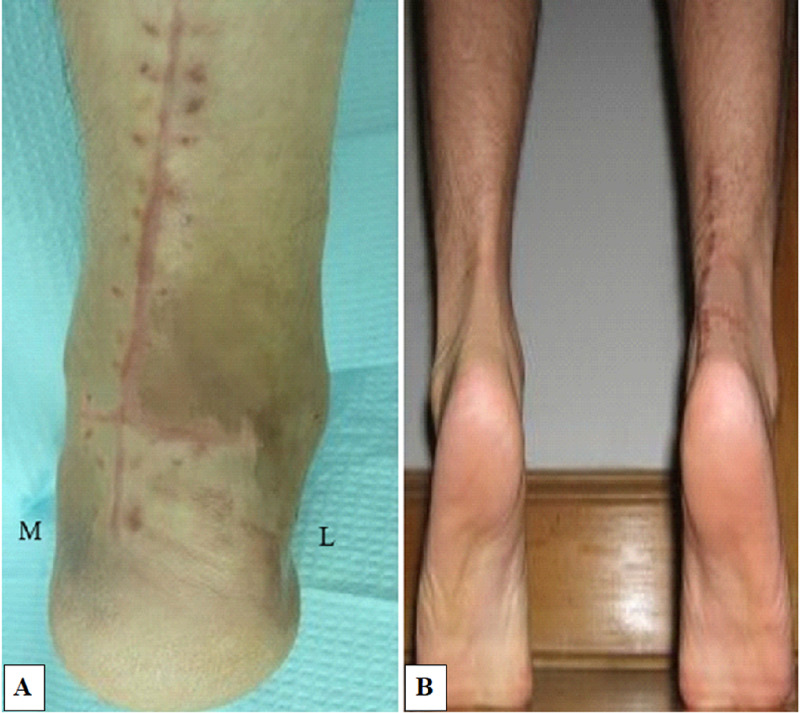
Follow-up images (A) At six weeks, the patient’s wound had healed well but a hypertrophic scar was noted; (B) At the one-year follow-up, the patient had good power in the Achilles tendon and active plantar flexion was comparable to the opposite side M-medial; L-lateral

An above-knee cast with the knee in 30 degrees of flexion and the ankle in gravity equinus was given for six weeks followed by gradual weight-bearing and range of motion exercises. The postoperative period was uneventful but a hypertrophic scar was observed at the wound site at six weeks (Figure 3A). At the end of one year, the patient could walk full weight-bearing with good power in plantar-flexion and had ankle movements comparable to the opposite side (Figure 3B). 

## Discussion

Uttarayan is the season of kite flying in India. Flying kites from rooftops, using dangerous kite-lines (manja), and chasing stray kites are common activities during this festival. Statistics about injuries and deaths caused due to kite flying in India and other South Asian countries like Bangladesh and Pakistan are scarce [1]. However, similar cases have been reported from time to time [4-6].

Recently, manja made of polypropylene thread (nylon) called ‘Chinese Manja’ (because it was imported from China) became very popular due to its increased strength, which provided an edge to kite-flying competitors. It was later banned as it proved to be very hazardous to both humans and animals, in particular the birds [1]. Numerous casualties and some fatalities from falls, deep-cut injuries (like throat cuts), road accidents while chasing kites, and electrocution are known to occur in flyers, two-wheeler riders, and pedestrians [4-6]. The severity of the injury depends on the speed of the vehicle and kite, which is directly related to wind speed [1,4]. It is important to understand the mechanism, pathology, medico-legal aspect, and prevention of injuries caused by manja. Wankhede described some characteristic features of manja injuries like they are almost always oriented transversely along the contour of the body, they are usually unidirectional and single, wound edges are abraded, depth is maximum at the center of the wound, and glass particles can be found at the angle of the injury in the direction opposite to the sliding manja [1].

The management of an incised wound caused by manja is similar to that of any other incised wound. A careful neurovascular examination is warranted because the depth and damage are maximum at the center and deeper injuries may be missed by an unsuspecting eye, which may see the wound as just a clean superficial laceration [1,3]. Thorough and gentle lavage with at least four to five liters of normal saline is a must because the wound often contains fine glass particles that stick to the tissues as the thread sinks deeper while cutting [1,3].

It is generally accepted that surgical repair should be performed in acute open tendoachilles ruptures [7]. However, a primary treating physician may miss a tear and just do a simple skin closure. The injury then gets neglected and may later present as a chronic rupture. Chronic ruptures should be reconstructed or repaired (with or without augmentation) in young and physically active patients, whereas in older patients, surgery may not be performed, as the results of conservative treatment are acceptable in sedentary lifestyles [7].

A manja injury to a kite-flyers' hand can be prevented by wearing gloves. Wearing full clothes prevents injuries to other parts of the body. To avoid manja injuries to pedestrians and two-wheeler riders, kite flying should be permitted only at designated locations during festivals [1]. These sites should be away from town and traffic and not located near an airport, electric service line, or bird sanctuary [1]. Agencies related to administration, games and sports, and traffic police should devise, pass, and implement rules and regulations after joint efforts.

## Conclusions

Public awareness should be created among kite flyers and passers-by, explaining the risks and hazards related to kite flying and highlight the precautionary steps that can be taken. There is a need to revisit and emphasize the hazards of manja, as they hold medico-legal and social importance. It is wise to suspect and check for associated neurovascular damage in such injuries because if neglected, the patient may suffer from increased morbidity and loss of productivity.

## References

[1] Wankhede AG, Sariya DR (2008). “Manja” - a dangerous thread. J Forensic Leg Med.

[2] Järvinen TA, Kannus P, Maffulli N, Khan KM (2005). Achilles tendon disorders: etiology and epidemiology. Foot Ankle Clin.

[3] Bagaria V (2015). Achilles tendon rupture secondary to kite string (manja) injury: a rare etiology seen in two cases. J Foot Ankle Surg.

[4] Ventura J, Hirano IES, Fraga GP II (2011). Glass-coated kites and cervical injuries: a serious threat to children and adults. Clinics.

[5] Tiwari VK, Sharma D (1999). Kite-flying: a unique but dangerous mode of electrical injury in children. Burns.

[6] Neto JB, Ferreira GC, Filho AL, Fontes MO, Bomfim F, Abrantes WL (2000). Kiting injuries: report of two cases and discussion. J Trauma.

[7] Järvinen TA, Kannus P, Paavola M, Järvinen TL, Józsa L, Järvinen M (2001). Achilles tendon injuries. Curr Opin Rheumatol.

